# Salvage Surgery Following Systemic Therapy in Initially Unresectable Non‐Small Cell Lung Cancer

**DOI:** 10.1111/1759-7714.70209

**Published:** 2025-12-17

**Authors:** Katsutoshi Seto, Yoshitsugu Horio, Katsuhiro Masago, Eiichi Sasaki, Hiroaki Kuroda, Yuta Matsubayashi, Hisanori Iwashimizu, Osamu Noritake, Keiyu Sato, Takuya Matsui, Soichiro Suzuki, Junichi Shimizu, Yutaka Fujiwara, Noriaki Sakakura

**Affiliations:** ^1^ Department of Thoracic Surgery Aichi Cancer Center Hospital Nagoya Japan; ^2^ Department of Thoracic Oncology Aichi Cancer Center Hospital Nagoya Japan; ^3^ Department of Pathology and Molecular Diagnostics Aichi Cancer Center Hospital Nagoya Japan; ^4^ Department of Thoracic Surgery Teikyo University Mizonokuchi Hospital Kawasaki Japan

**Keywords:** immunotherapy, non‐small cell lung cancer, overall survival, salvage surgery, systemic therapy

## Abstract

**Background:**

This study evaluated the safety, long‐term survival, and prognostic factors associated with salvage pulmonary surgery following systemic therapy for initially unresectable non‐small cell lung cancer (NSCLC).

**Methods:**

Between 2014 and 2024, this single‐center retrospective review identified 32 patients (median age: 61.0 years) with NSCLC initially considered unresectable who subsequently underwent curative‐intent pulmonary resection after chemotherapy, targeted therapy, and/or immunotherapy. The primary endpoint was overall survival (OS); secondary endpoints included recurrence‐free survival (RFS), major morbidity (Clavien–Dindo grade ≥ IIIa), and R0 resection rate. The Kaplan–Meier method and log‐rank test were employed.

**Results:**

Reasons for unresectability at initial diagnosis were distant metastasis (*n* = 20; 62.5%), N3 nodal disease (*n* = 6; 18.8%), bulky N2 nodal disease (*n* = 3; 9.4%), and tumor or nodal extension requiring pneumonectomy (*n* = 3; 9.4%). Overall, 65.6% patients underwent lobectomy, with R0 resection achieved in 81.3% and pathological complete or major response observed in 15.6%. Overall complication and major morbidity rates were 12.5% and 3.1%, respectively; no 90‐day mortality was observed. After a median follow‐up of 40.1 months, median OS was not reached, whereas median RFS was 49.9 months; 5‐year OS and RFS were 75.0% (95% CI 51.6–88.3) and 46.3% (95% CI 26.3–64.2), respectively. Notably, adenocarcinoma histology was significantly more prevalent in the good‐prognosis group (88.9% vs. 35.7%, *p* = 0.003).

**Conclusions:**

Salvage pulmonary surgery following systemic therapy is safe, yielding a 5‐year OS rate of 75% in carefully selected patients with advanced NSCLC. Prevalent adenocarcinoma histology in the good‐prognosis cohort is associated with superior outcomes.

## Graphical abstract

Salvage pulmonary resection after systemic therapy for initially unresectable non‐small cell lung cancer achieved a high R0 rate with minimal morbidity and no perioperative mortality. Careful multidisciplinary selection facilitated a durable 5‐year survival rate of 75%. These findings highlight the feasibility and oncological benefit of surgery in the immunotherapy era.

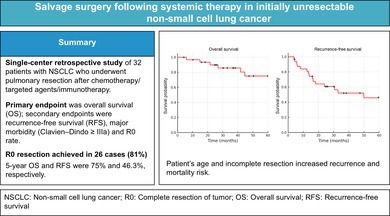

## 1 Introduction

Systemic therapy for non‐small cell lung cancer (NSCLC) has markedly improved patient outcomes, particularly with the advent of molecular‐targeted agents—such as epidermal growth factor receptor (*EGFR*) tyrosine kinase inhibitors (TKIs) and anaplastic lymphoma kinase (*ALK*) inhibitors—and immune checkpoint inhibitors (ICIs), in addition to conventional platinum‐based cytotoxic chemotherapy [1, 2, 3]. Given the high response rates and sustained efficacy of these regimens, an increasing number of cases have been reported in which loco‐regional lesions become substantially reduced or localized following definitive non‐surgical treatment, even in patients with advanced NSCLC initially considered unresectable. In such cases, “salvage surgery”—a curative‐intent resection targeting residual or re‐enlarging disease—has been performed with increasing frequency [4, 5].

As salvage surgery is undertaken following systemic pharmacotherapy, it may present unique perioperative challenges, including therapy‐induced fibrosis and immune‐mediated tissue alterations [6, 7, 8, 9]. Several retrospective studies have reported outcomes comparable to those achieved with induction chemotherapy followed by surgery [10], and 5‐year overall survival (OS) rates exceeding 50% after salvage surgery have also been described [4, 11], suggesting that the procedure may confer a prognostic benefit. Nevertheless, the available data remain limited and robust evidence regarding the true impact of salvage surgery on long‐term survival has yet to be established.

Therefore, this retrospective study analyzed cases of NSCLC treated with salvage surgery at our institution to evaluate the perioperative safety, oncological outcomes, and clinical significance of salvage surgery. We hypothesized that (1) salvage surgery can be safely performed after systemic therapy with acceptable major complication and short‐term mortality rates, and that (2) salvage surgery in selected patients is associated with favorable long‐term outcomes (recurrence‐free survival [RFS]/OS).

## 2 Methods

### 2.1 Study Design and Patients

**FIGURE 1:**
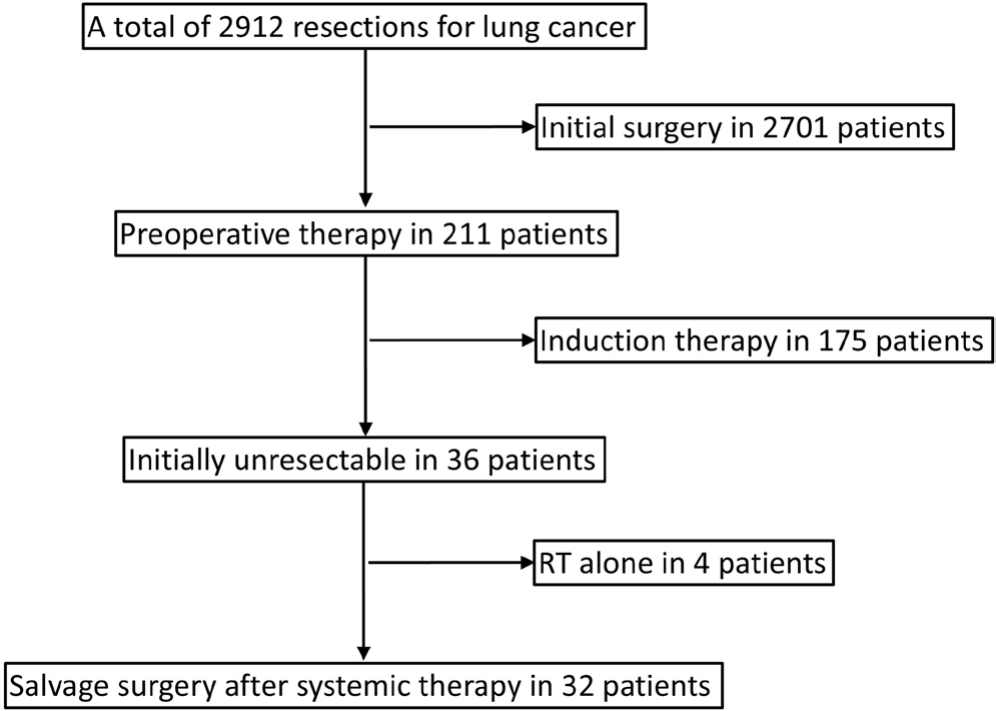
Patient flowchart. The counts refer to cases recorded in the surgical database only. Salvage surgery refers to curative‐intent resection after systemic therapy in patients with initially unresectable non‐small cell lung cancer. Induction therapy refers to preoperative therapy for initially resectable cases. RT, radiotherapy.

This single‐center retrospective observational study was conducted at the Aichi Cancer Center. Among 2912 pulmonary resections performed between 1 January 2014 and 31 December 2024, we identified 32 patients who underwent salvage surgery. Figure 1 shows the patient selection flowchart and reasons for exclusion. Salvage surgery was defined as curative‐intent resection after systemic therapy in patients initially deemed unresectable owing to at least one of the following criteria: (i) distant metastasis (M1); (ii) inoperable mediastinal nodal disease—specifically cN3 or bulky/multi‐station N2; or (iii) a primary tumor whose anticipated extent would necessitate pneumonectomy. Single‐station, non‐bulky N2 disease was managed within a planned induction‐therapy pathway and was therefore excluded from this definition. Patients treated with radiotherapy (RT) alone were ineligible because RT was regarded as a local treatment; concurrent chemoradiotherapy (CRT) was considered to include systemic therapy and thus met the eligibility criteria. Surgical candidacy was determined at a multidisciplinary team (MDT) meeting (thoracic surgery, thoracic oncology, radiation oncology, and diagnostic radiology), and the absence of new or progressive distant metastases at surgery was required. At the time of salvage resection, patients with measurable or progressive distant metastases were excluded. Oligometastatic disease was considered eligible only if all metastatic sites had been definitively controlled by prior local therapy (e.g., stereotactic radiotherapy, ablation, or resection) and no measurable disease remained at operation. Consequently, no patient had radiologically measurable distant disease at the time of surgery. Within the surgical database, screening for the predefined salvage‐eligibility criteria yielded the analytic cohort after excluding records with definition violations, non‐curative intent, missing primary outcomes, or duplicate entries. Procedures that ultimately resulted in microscopic (R1) or macroscopic (R2) residual disease were retained for outcome assessment. The sample size for this study was determined by including all consecutive salvage surgeries performed at our institution during the study period; no a priori sample size calculation was performed. At the time of surgery, disease was confined to an intrapulmonary lesion and/or to hilar or mediastinal lymph nodes.

### 2.2 Surgical Strategies

Operations were undertaken with curative intent. Lobectomy was the default procedure, with segmentectomy, wedge resection, or extended resection selected according to tumor location, bronchovascular anatomy, and predicted postoperative pulmonary function. In particular, segmentectomy or wedge resection was permitted with the aim of achieving local control and preserving pulmonary function in patients who presented with stage IV disease or metastases outside the field of systematic lymph node dissection and those with only primary lesion localization after systemic therapy. When R0 resection was considered to be difficult, bronchoplastic (sleeve) procedures, pulmonary artery reconstruction (PA plasty), or en bloc chest‐wall resection were employed. Pneumonectomy was not excluded a priori and was considered when R0 could not be achieved by alternative procedures, and postoperative function was anticipated to be acceptable. The surgical approach—open thoracotomy, thoracoscopic, or robot‐assisted—was chosen after the comprehensive appraisal of prior irradiation, degree of inflammatory fibrosis, need for reconstruction, likelihood of chest‐wall invasion, and extent of adhesions. Patient safety was prioritized; if dissection planes were lost, the operative field was inadequate, or bleeding risk increased, conversion to thoracotomy was undertaken at a low threshold. In cases after CRT or when chest‐wall invasion was strongly suspected, primary thoracotomy was generally selected. Margin management comprised intraoperative frozen‐section assessment of the bronchial stump, pulmonary arterial and venous transection margins, and chest‐wall resection margins whenever feasible; if malignant involvement was suspected, additional resection was performed to achieve R0. In patients after radiotherapy or with pronounced inflammatory fibrosis, intercostal muscle‐flap (or similar) coverage was liberally applied in view of potential hypoperfusion and delayed healing at anastomoses and stumps. Systematic lymph‐node dissection (ND2a‐1/ND2a‐2) was performed as a rule, with prudent limitation in heavily fibrotic cases after CRT to avoid nerve or vascular injury.

### 2.3 Postoperative Adjuvant Therapy

The indication for, and content of, postoperative adjuvant therapy (systemic therapy and/or postoperative radiotherapy) were determined for all cases at an MDT meeting. Decisions were based on pathological findings (yp stage, R status, extranodal extension, pathological treatment response), general condition (performance status), comorbidities, postoperative complications, and residual toxicities, imaging findings, and patient preference.

### 2.4 Data Collection

Clinical data were extracted from electronic medical records and operative reports. Patient characteristics included age, sex, smoking history, performance status, and comorbidities. Tumor‐related variables included histological subtype, driver mutations such as EGFR and ALK, and clinical stage according to the 8th edition of the cTNM classification [12]. Systemic‐therapy data included regimens, number of treatment lines, and response by RECIST v1.1 [13]. Surgical variables included type of resection, surgical approach (thoracotomy/thoracoscopic/robotic), operative time, estimated blood loss, and intraoperative transfusion. Pathology included pTNM (8th edition) [12], pathological complete response (pCR) or major pathological response (MPR), and resection margin status (R0/R1/R2). Peri‐operative data comprised the length of stay, complications graded by the Clavien–Dindo classification, and 90‐day mortality. Follow‐up captured recurrence site and timing, post‐recurrence treatment, and vital status at last contact.

### 2.5 Pathological Assessment

Pathological response was evaluated on hematoxylin–eosin‐stained sections. pCR was defined as 0% residual viable tumor (RVT) in both the primary lesion and all examined lymph nodes, and MPR as ≤ 10% RVT [14].

### 2.6 Outcomes

The primary outcome was OS, defined from the date of salvage surgery to death from any cause. Secondary outcomes were RFS, the incidence of serious postoperative complications within 30 days (Clavien–Dindo grade ≥ IIIa), and the R0 resection rate. RFS was defined from surgery to first documented recurrence or death, whichever occurred first.

### 2.7 Statistical Analyses

As this study was exploratory and descriptive in nature with complete enrollment of consecutive cases, sample size planning based on a priori effect size assumptions or power analyses was not performed. Continuous variables are reported as medians with interquartile ranges, and categorical variables as frequencies and percentages. OS and RFS were estimated using the Kaplan–Meier method and compared, where appropriate, with the log‐rank test. Early recurrence was defined as relapse within 24 months and early death as all‐cause mortality within 24 months; patients meeting either criterion were classified as the poor‐prognosis group. The 24‐month threshold was prespecified to balance group sizes in this small sample and to minimize instability of estimates. Categorical variables were compared using the χ^2^ test (or Fisher's exact test when expected counts were < 5). Two‐sided *p* values of < 0.05 were considered statistically significant. Analyses were conducted using EZR (Easy R) version 1.61 (Saitama Medical Center, Jichi Medical University, Saitama, Japan), a graphical user interface for R Commander facilitating survival and competing‐risk analysis without command‐line input [15].

### 2.8 Ethical Considerations

Given the retrospective design and the use of de‐identified data, the requirement for written informed consent was waived; an opt‐out procedure was implemented whereby study information was publicly disclosed and patients were afforded the opportunity to decline participation. The protocol was approved by the Ethics Review Board of the Aichi Cancer Center (approval no. 2025‐0‐130).

## 3 Results

### 3.1 Patient Background and Tumor Characteristics

A total of 32 patients underwent salvage surgery during the study period. The median age was 61.0 years (interquartile range [IQR] 50.5–71.5), and 21 patients (65.6%) were men. The median Brinkman Index was 392.5 (IQR 0–1005). Eastern Cooperative Oncology Group (ECOG) PS was 0 in 30 patients and 1 in two patients; no patient had a PS ≥ 2. Histologically, 21 tumors (65.6%) were adenocarcinomas, seven (21.9%) were squamous cell carcinomas, and four (12.5%) had other histologies. *EGFR* mutations were identified in 10 patients (31.3%), *ALK* fusions in two (6.3%), ROS proto‐oncogene 1 (*ROS1*) fusions in two (6.3%), and a rearranged during transfection (*RET*) fusion in one (3.1%). Reasons for unresectability at initial diagnosis were as follows: distant metastasis (*n* = 20; 62.5%), N3 nodal disease (*n* = 6; 18.8%), bulky N2 nodal disease (*n* = 3; 9.4%), and tumor or nodal extension requiring pneumonectomy (*n* = 3; 9.4%). The median number of systemic therapy lines administered prior to surgery was one. Cytotoxic chemotherapy was administered in 71.9% of patients, molecular‐targeted therapy in 43.8%, and ICIs in 37.5% (Table 1). The distribution of preoperative systemic regimens is summarized in Table S1.

**TABLE 1:** Patient background and tumor characteristics.

Variable (*n* = 32)	No. of patients (%) or Median (range)
Age	61.0 (27–80)
Sex	
Men	21 (65.6)
Women	11 (34.4)
Brinkman index	392.5 (0–2520)
Performance status	
0	30 (93.7)
1	2 (6.3)
Histology	
Adenocarcinoma	21 (65.6)
Squamous cell carcinoma	7 (21.9)
Others	4 (12.5)
Driver mutation	
*EGFR*	10 (31.3)
*ALK*	2 (6.3)
*ROS1*	2 (6.3)
*RET*	1 (3.1)
Negative	17 (53.1)
Initial clinical stage	
II	1 (3.1)
III	11 (34.4)
IV	20 (62.5)
Reason for unresectability	
Distant metastases (M1)	20 (62.5)
N3	6 (18.8)
Bulky N2	3 (9.4)
Pneumonectomy required	3 (9.4)
Preoperative therapy	
Systemic therapy lines	1 (1–6)
Cytotoxic therapy	23 (71.9)
Molecular‐targeted therapy	14 (43.8)
ICI	12 (37.5)

Abbreviations: *ALK*, anaplastic lymphoma kinase; *EGFR*, epidermal growth factor receptor; ICI, immune checkpoint inhibitor; *RET*, rearranged during transfection; *ROS1*, ROS proto‐oncogene 1.

### 3.2 Surgery‐Related Parameters and Pathological Findings

Lobectomy was performed in 21 patients (65.6%), segmentectomy in six (18.8%), and wedge resection in five (15.6%). Pneumonectomy was not performed in this cohort. Furthermore, neither bronchoplastic (sleeve) procedures nor pulmonary artery reconstructions were performed in this cohort (both 0/32, 0%). A thoracoscopic approach was used in 13 cases (40.6%) and thoracotomy in 19 cases (59.4%); no robot‐assisted procedures were performed. The median operative time was 226.5 min (IQR 178.5–264.3), and the median estimated blood loss was 30 mL (IQR 5–121.3); only one patient (3.1%) required an intraoperative transfusion. Pathologically, the tumor stage was 0 in four patients (12.5%), I in 14 (43.8%), II in seven (21.9%), III in four (12.5%), and IV in three (9.4%). pCR was achieved in four patients (12.5%) and MPR in five (15.6%). R0 resection was achieved in 26 patients (81.3%), while R1 and R2 resections were performed in five patients (15.6%) and one patient (3.1%), respectively (Table 2). Non‐R0 resections were distributed as follows: three patients with pathological detection of pleural dissemination, one patient with a positive bronchial margin (negative result in intraoperative frozen section analysis), one patient with a positive dissection margin of the mediastinal pleura, and one patient with a positive dissection margin of the diaphragm. All cases diagnosed as stage IV by postoperative pathology showed pleural dissemination.

**TABLE 2:** Surgery‐related parameters and pathological findings.

	No. of Patients (%)
Surgical procedure	
Lobectomy	21 (65.6)
Segmentectomy	6 (18.8)
Wedge resection	5 (15.6)
Combined resection	
Chest wall	3 (9.4)
Parietal pleura	3 (9.4)
Others	1 (3.1)
Surgical approach	
VATS	13 (40.6)
Open	19 (59.4)
R0 resection	26 (81.3)
Pathological stage	
0	4 (12.5)
I	14 (43.8)
II	7 (21.9)
III	4 (12.5)
IV	3 (9.4)
Pathological response	
pCR	4 (12.5)
MPR	5 (15.6)

Abbreviations: MPR, major pathological response (≤ 10% residual viable tumor); pCR, pathological complete response (0% residual viable tumor); VATS, video‐assisted thoracic surgery.

### 3.3 Perioperative Complications and Length of Stay

Detailed patient‐level perioperative outcomes and complications are presented in Table S2. The overall complication rate was 12.5% (4/32 patients). One patient (3.1%) experienced a Clavien–Dindo grade IIIa complication, specifically a prolonged air leak requiring pleurodesis. The median postoperative hospital stay was 5.0 days (IQR 2.8–8.0). No deaths occurred within 90 days of surgery (Table 3).

**TABLE 3:** Perioperative complications and postoperative outcomes.

	No. of Patients (%) or Median (range)
Postoperative complication (any grade)	4 (12.5)
Postoperative complication (Clavien–Dindo grade ≥ IIIa)	1 (3.1)
Prolonged air leak	1 (3.1)
Recurrent nerve palsy	1 (3.1)
Chest pain	1 (3.1)
Atrial fibrillation	1 (3.1)
Postoperative outcomes	
Postoperative hospital stay (days)	5 (2–21)
90‐day mortality	0 (0)

### 3.4 Post‐Operative Adjuvant Therapy and Post‐Recurrence Treatment

Thirteen of the 32 patients (41%) received systemic adjuvant therapy before evidence of recurrence. The regimens employed—along with the treatment administered—are listed in Table S3.

### 3.5 Survival Analysis

**FIGURE 2:**
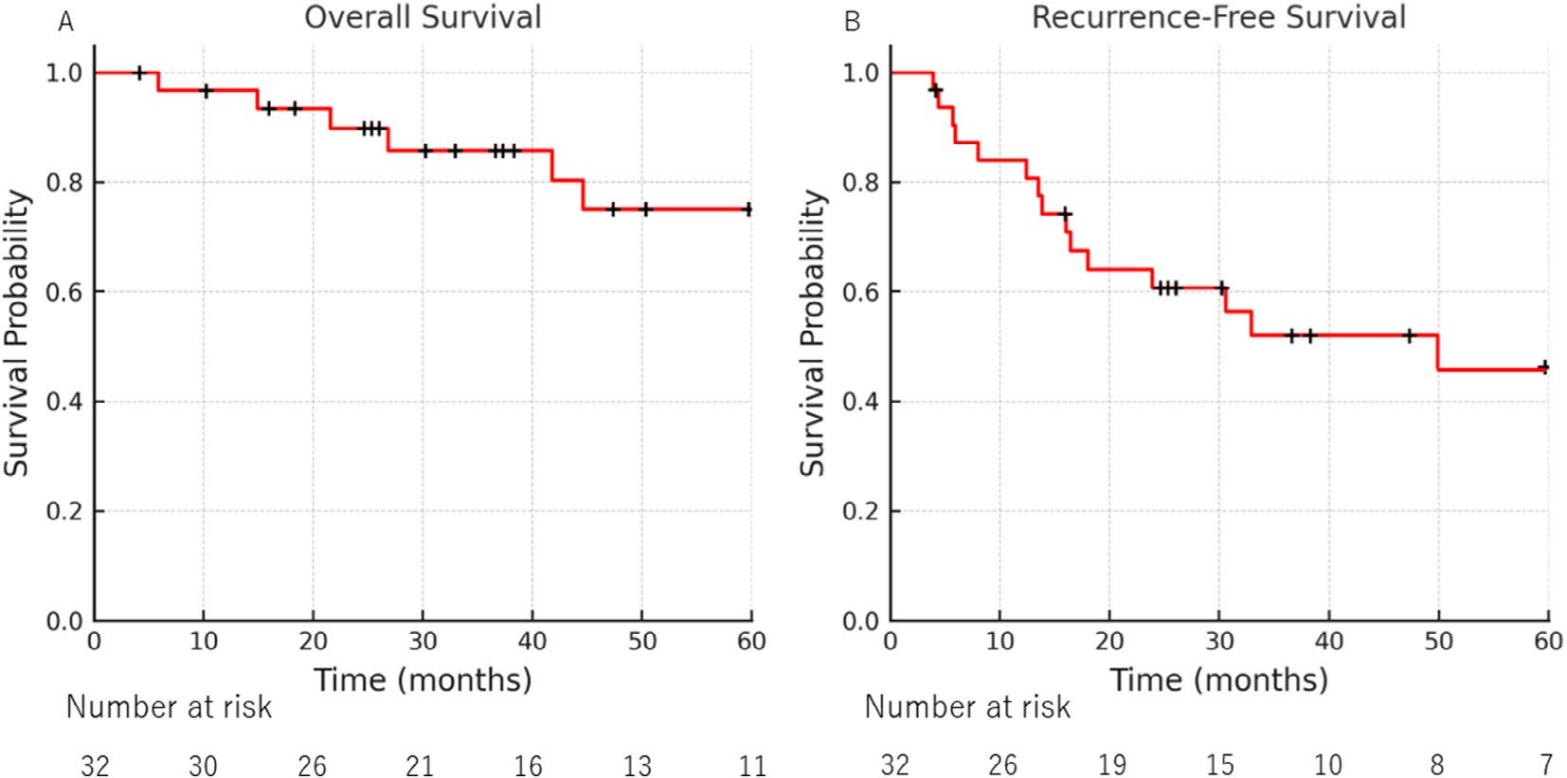
Kaplan–Meier survival after salvage pulmonary resection for initially unresectable or metastatic non–small cell lung cancer (NSCLC). (A) Overall survival (OS). Median OS was not reached at a median follow‐up of 40.1 months. The 1‐, 3‐, and 5‐year OS rates were 96.8%, 85.8%, and 75.0%, respectively. (B) Recurrence‐free survival (RFS). Median RFS was 49.9 months (95% confidence interval [CI] 26.3–64.2). The 1‐, 3‐, and 5‐year RFS rates were 84.0%, 52.1%, and 46.3%, respectively.

The median follow‐up duration was 40.1 months (IQR 25.1–69.5). The Kaplan–Meier curve for OS is presented in Figure 2A. Median OS was not reached. The 1‐, 3‐, and 5‐year OS rates were 96.8%, 85.8%, and 75.0% (95% CI 51.6%–88.3%), respectively. The RFS curve is shown in Figure 2B. Median RFS was 49.9 months (95% CI 16.4–Not Available [N/A]), with 1‐, 3‐, and 5‐year RFS rates of 84.0%, 52.1%, and 46.3% (95% CI 26.3–64.2), respectively.

### 3.6 Predictors of Outcome in the Expanded Two‐Group Comparison

As shown in Table 4, adenocarcinoma histology remained the sole factor significantly associated with a favorable prognosis (88.9% vs. 35.7%; *p* = 0.003). Absence of prior ICI therapy (77.8% vs. 42.9%; *p* = 0.068) and age < 65 years (72.2% vs. 42.9%; *p* = 0.149) again demonstrated non‐significant trends, while none of the other clinicopathological variables—including driver‐mutation status, sex, smoking exposure, ECOG performance status, initial Stage IV disease, R0 resection, or pathological response—showed a statistically significant association.

**TABLE 4:** Clinicopathological factors according to prognosis group.

Factor	Good prognosis	Poor prognosis	*p* value^a^
	(*n* = 18)	(*n* = 14)	
Adenocarcinoma histology	16/18 (88.9%)	5/14 (35.7%)	0.003
Absence of prior ICI therapy	14/18 (77.8%)	6/14 (42.9%)	0.068
Age < 65 years	13/18 (72.2%)	6/14 (42.9%)	0.149
Driver mutation present (*EGFR/ALK/ROS1/RET*)	10/18 (55.6%)	4/14 (28.6%)	0.165
Male sex	11/18 (61.1%)	10/14 (71.4%)	0.712
Current or former smoker	9/18 (50.0%)	9/14 (64.3%)	0.490
ECOG PS ≥ 1	0/18 (0%)	2/14 (14.3%)	0.183
Stage IV at diagnosis	12/18 (66.7%)	8/14 (57.1%)	0.718
R0 resection	15/18 (83.3%)	11/14 (78.6%)	1.000
pCR or MPR	4/18 (22.2%)	1/14 (7.1%)	0.355

Abbreviations: *ALK*, anaplastic lymphoma kinase; ECOG, eastern cooperative oncology group; *EGFR*, epidermal growth factor receptor; ICI, immune checkpoint inhibitor; MPR, major pathological response (≤ 10% residual viable tumor); pCR, pathological complete response (0% residual viable tumor); *RET*, rearranged during transfection; *ROS1*, ROS proto‐oncogene 1.

^a^ *p*‐values were calculated with Fisher's exact test.

### 3.7 Recurrence Patterns

During the follow‐up period, disease recurrence was observed in 14 of the 32 patients (43.8%). Recurrence patterns were as follows: local or regional recurrence in nine patients (28.1%), distant recurrence in four patients (12.5%), and concurrent local and distant recurrence in one patient (3.1%). Distant recurrences were located in the brain (*n* = 3) and bone (*n* = 1) (Figure S1).

## 4 Discussion

In this retrospective study of 32 patients with initially unresectable or metastatic NSCLC, salvage pulmonary surgery following systemic therapy demonstrated both technical safety and oncological efficacy. Postoperative morbidity was limited to 12.5% with no 90‐day mortality, and a median hospital stay of only five days, underscoring the procedure's tolerability. Notably, R0 resection was achieved in 81% of patients, and MPR was observed in 15.6%. These surgical outcomes translated into favorable long‐term results: the median OS was not reached at a median follow‐up of 40 months, while the 5‐year OS and RFS rates were 75% and 46%, respectively. Although 44% of patients experienced recurrence, the distribution was balanced between locoregional and distant sites. The relatively high proportion of locoregional relapse reflects our selection criteria, which excluded cases showing new or progressive distant disease between the completion of systemic therapy and surgery.

Even in patients initially deemed unresectable, several studies have concluded that salvage surgery is feasible if systemic and local therapies achieve adequate control of both the primary tumor and metastatic lesions under strict selection criteria [4, 5, 11, 16, 17, 18, 19, 20, 21, 22]. In terms of safety, previous reports have documented postoperative complication rates ranging from 11.1% to 51.0% [4, 11, 16, 19, 20, 21, 22, 23], whereas the present study's complication rate was at the lower end of this range. Similarly, the 90‐day mortality rate was 0%, which compares favorably with the 0%–5.7% reported in earlier studies [4, 11, 16, 19, 22]. These outcomes suggest effective optimization of preoperative general condition through careful multidisciplinary evaluation and patient selection. Approximately 40% of salvage surgeries in our cohort were performed using video‐assisted thoracoscopic surgery (VATS), a minimally invasive approach that may have facilitated postoperative recovery and contributed to the low complication rate. Collectively, these safety findings support the notion that salvage surgery is a well‐tolerated therapeutic strategy for NSCLC when undertaken by an experienced surgical team in carefully selected patients. Consistent with our results, high‐complexity reconstructions—namely bronchoplasty and pulmonary artery reconstruction—were not undertaken in this cohort. Given that the candidates originated from a population initially deemed inoperable, MDT deliberations may have favored a safety‐first operative strategy and avoided aggressive reconstruction in cases where complete resection could be achieved without it.

Five‐year OS rates reported in the literature vary widely, ranging from 20% to 75.1% [3, 4, 5, 16, 17, 19, 21, 22, 23, 24]. The 5‐year OS rate observed in the present study lies at the upper end of this spectrum. Although our cohort was heterogeneous, comprising patients who received conventional cytotoxic chemotherapy, molecular‐targeted agents, ICIs, or various combinations thereof prior to salvage surgery, the findings suggest a substantial survival benefit in those whose primary and metastatic lesions were adequately controlled preoperatively. MPR was achieved in 15.6% of patients; this rate was comparable to those reported by Suzuki et al. [22] and Lin et al. [24] but lower than those observed by Mohamed et al. [18] and Guerrera et al. [4]. Nevertheless, the high R0 resection rate and favorable OS outcomes in our cohort indicate that long‐term survival can be significantly improved when complete macroscopic resection is achieved following adequate tumor downsizing and metastatic control, even in the absence of MPR or pCR.

The advent of next‐generation molecularly targeted therapies, such as TKIs for *EGFR, ALK, ROS1*, and *RET* mutations, has resulted in markedly improved PFS and OS compared to those with conventional chemotherapy. Recent trials, including the phase III CROWN trial (5‐year PFS 60%) and a pivotal phase II study (5‐year OS 76% with lorlatinib), highlight this survival benefit [25, 26]. In our cohort, two *ALK*‐positive and two *ROS1‐*positive patients underwent salvage surgery after durable responses to targeted therapy and have remained recurrence‐free for more than 30 months. These observations suggest that, in oncogene‐addicted tumors with sustained systemic control, surgical consolidation of residual intrathoracic disease may provide a path toward long‐term remission—or even cure—when integrated into a multimodal strategy.

The concept of “adjuvant lung surgery” has recently been introduced and refers to the planned resection of residual intrathoracic disease following systemic therapy that has rendered previously unresectable tumors—typically stage IIIb or IV with distant or locally advanced involvement—sufficiently controlled, thereby complementing the systemic treatment [27]. In contrast to salvage surgery, which represents an unplanned rescue procedure, adjuvant surgery is an elective intervention embedded in a multimodal strategy from the outset. Its objectives are to reduce the residual tumor burden, eradicate therapy‐resistant clones, and obtain high‐quality tissue to guide subsequent precision therapies. Some cases in our cohort resembled adjuvant lung surgery, where systemic therapy converted an unresectable tumor to resectable status, followed by surgery, within multimodal management. It should be noted, however, that the surgical indications in this study were not governed by a rigorously predefined adjuvant protocol but were instead determined on a case‐by‐case clinical basis.

Adenocarcinoma emerged as the only statistically significant favorable prognostic factor, reflecting its intrinsically slower growth kinetics and the availability of multiple genotype‐matched targeted agents (*EGFR, ALK, ROS1*, and *RET* inhibitors), which enable effective sequential treatment following recurrence and thereby may extend OS. Patients who underwent induction therapy without ICIs tended to have a better prognosis than those who received ICIs (77.8% vs. 42.9%; *p* = 0.068, Good = 18, Poor = 14). This difference may be attributable to treatment‐selection bias, with high‐risk, PD‐L1‐high tumors more likely to receive early ICI therapy, and the enrichment of “responsive” cases among the ICI‐naïve cohort. Given the limited sample size and a *p*‐value above the conventional threshold, this trend did not reach statistical significance, and ICI‐naivety cannot currently be considered an independent predictor.

In oligoprogressive advanced lung cancer, several focal therapies—including stereotactic radiotherapy, proton‐beam therapy, radiofrequency ablation, and cryoablation—are clinically available. Nevertheless, surgical salvage resection offers distinct advantages that non‐excisional modalities cannot provide. First, it allows macroscopic removal of the lesion and pathologic confirmation of the R0 margin on the resected specimen, whereas non‐surgical approaches rely on radiologic shrinkage or disappearance and cannot fully exclude microscopic residual disease. Second, salvage surgery permits systematic lymph‐node dissection, thereby eradicating occult nodal metastases and enabling accurate pathologic up‐ or down‐staging. Third, acquisition of the surgical specimen makes it possible to examine, at the microscopic level, the tumor's histologic response to prior systemic therapy and the emergence of resistant clones. Such pathologic and molecular information not only guides postoperative selection or modification of targeted agents and ICIs, but also serves as evidence for future individualized therapy. Although the risk of surgical morbidity cannot be ignored, the favorable perioperative outcomes demonstrated in this study indicate that, in appropriately selected and physiologically fit patients, salvage surgeries should be actively considered among these treatment options.

This study had some limitations. First, it was a single‐center, retrospective study with a small sample (*n* = 32) and few events; thus, the statistical power was limited and robust multivariable adjustment was precluded. Moreover, the study included a surgically ascertained cohort derived from a surgical database, and we could not enumerate patients managed exclusively by medical therapy and those who, after systemic therapy, were referred to the thoracic surgery department but ultimately did not undergo resection. Consequently, channeling/selection bias cannot be quantitatively assessed, and the observed outcomes may appear selectively favorable. Second, the cohort exhibited substantial oncological heterogeneity; specifically, there were variations in the initial reason for unresectability (M1, cN3, bulky/multistation N2, anticipated pneumonectomy), driver alterations, preoperative treatment modality and line, and exposure to immune checkpoint inhibitors. Third, the absence of a contemporaneous non‐surgical control group prevented causal inference regarding the effect of salvage surgery on long‐term outcomes. Fourth, because this retrospective study was based on a single‐center surgical database, external validity and reproducibility were limited. Thus, external validation in multicenter cohorts with prospectively standardized data collection is warranted. Taken together, these limitations preclude definitive conclusions regarding the safety of salvage surgery. Thus, our observations should be viewed as hypothesis‐generating and require validation in multicenter, prospectively standardized cohorts or rigorously adjusted comparative studies.

In summary, this study demonstrates the safety and efficacy of salvage surgery following systemic therapy in patients with NSCLC initially assessed as unresectable or metastatic. The perioperative complication rate was 12.5%, the 90‐day mortality rate was 0%, and R0 resection was achieved in 81% of cases. At a median follow‐up of 40.1 months, the 5‐year OS and RFS rates were 75% and 46%, respectively—figures that lie at the upper end of previously reported ranges. These findings support salvage surgery as a promising therapeutic strategy for extending survival in carefully selected patients. However, given the retrospective single‐center design and limited sample size, these results should be validated in future multicenter prospective studies.

## Author Contributions

**Katsutoshi Seto:** conceptualization, data curation, formal analysis, investigation, methodology, project administration, resources, software, visualization, writing – original draft, writing – review and editing. **Yoshitsugu Horio:** conceptualization, data curation, investigation, methodology, resources, supervision, validation, writing – review and editing. **Katsuhiro Masago:** data curation, formal analysis, investigation, resources, software, supervision, validation, writing – review and editing. **Eiichi Sasaki:** data curation, investigation, resources, writing – review & editing. **Hiroaki Kuroda:** investigation, supervision, validation, writing – review and editing. **Yuta Matsubayashi:** investigation, writing – review & editing. **Hisanori Iwashimizu:** investigation, writing – review and editing. **Osamu Noritake:** investigation, writing – review and editing. **Keiyu Sato:** investigation, writing – review and editing. **Takuya Matsui:** investigation, writing – review and editing. **Soichiro Suzuki:** investigation, writing – review and editing. **Junichi Shimizu:** investigation, writing – review and editing. **Yutaka Fujiwara:** investigation, writing – review and editing. **Noriaki Sakakura:** investigation, methodology, resources, supervision, validation, writing – review and editing.

## Funding

The authors have nothing to report.

## Ethics Statement

The protocol was approved by the Ethics Review Board of the Aichi Cancer Center (approval no. 2025‐0‐130).

## Consent

Given the retrospective design and the use of de‐identified data, the requirement for written informed consent was waived; an opt‐out procedure was implemented whereby study information was publicly disclosed and patients were afforded the opportunity to decline participation.

## Conflicts of Interest

Yutaka Fujiwara: Consultant/advisor: AstraZeneca, Chiome Bioscience, Daiichi‐Sankyo, Micron, Nippon Kayaku, Ono Pharmaceutical; Honoraria: AstraZeneca, Amgen, Bristol Myers Squibb, Chugai, Daiichi‐Sankyo, Eli Lilly, Johnson and Johnson, Merck Biopharma, Merck Sharp & Dohme, Novartis, Novocure, Ono Pharmaceutical, Pfizer, Takeda, Taiho Pharmaceutical; Institutional Research & Clinical Trial PI: Abbvie, Amgen, AnHeart, AstraZeneca, Astellas, Boehringer Ingelheim, Bristol Myers Squibb, Chugai, Eli Lilly, Incyte, Merck KGaA. Junichi Shimizu: Honoraria: Novartis, Daiichi‐Sankyo, Chugai, Taiho Pharmaceutical, MSD, AstraZeneca, Merck Biopharma, Takeda, Pfizer, Amgen, Ono Pharmaceutical. All other authors declare no conflicts of interest.

## Supporting information


**Table S1:** Systemic therapy regimens by patient.
**Table S2:** Perioperative outcomes and complications.
**Table S3:** Adjuvant therapies and post‐recurrence therapies.
**Figure S1:** Distribution of recurrence patterns after salvage surgery.

## Data Availability

De‐identified individual participant data that underlie the results reported in this article (text, tables, figures, and appendices) are available from the corresponding author upon reasonable request, subject to institutional and ethical approvals.
