# Supplementary material for: Salvage Surgery Following Systemic Therapy in Initially Unresectable Non‐Small Cell Lung Cancer
Source: Thorac Cancer. 2025 Dec 17;16(24):e70209. doi: 10.1111/1759-7714.70209 (PMC12709654; doi:10.1111/1759-7714.70209)
Supplement: Supplementary file 1 — Table S1: Systemic therapy regimens by patient. Table S2: Perioperative outcomes and complications. Table S3: Adjuvant therapies and post‐recurrence therapies. Figure S1: Distribution of recurrence patterns after salvage surgery. [file TCA-16-e70209-s001.docx]

**Table S1. Systemic Therapy Regimens by Patient**

| Patient_No. | Histology | Genetic Mutation | 1^st^ Line | 2^nd^ Line | 3^rd^ Line | 4^th^ Line | 5^th^ Line | 6^th^ Line | Total_Lines | Chemo_Used | Molecular-targeted therapy_Used | ICI_Used |
| --- | --- | --- | --- | --- | --- | --- | --- | --- | --- | --- | --- | --- |
| 1 | AD | EGFR | CDDP+VNR+RT | PEM | gefitinib |  |  |  | 3 | Yes | Yes | No |
| 2 | Sarcomatoid | - | CBDCA+PTX |  |  |  |  |  | 1 | Yes | No | No |
| 3 | AD | - | CDDP+VNR+RT |  |  |  |  |  | 1 | Yes | No | No |
| 4 | AD | ALK | CDDP+VNR+WBRT | CBDCA+PEM | ceritinib | PEM | alectinib | PEM | 6 | Yes | Yes | No |
| 5 | SQ | ALK | CDDP+GEM | crizotinib | DOC | ceritinib | alectinib |  | 5 | Yes | Yes | No |
| 6 | AD | EGFR | erlotinib |  |  |  |  |  | 1 | No | Yes | No |
| 7 | AD | EGFR | CDDP+VNR | gefiinib |  |  |  |  | 2 | Yes | Yes | No |
| 8 | AD | EGFR | gefitinib+PEM |  |  |  |  |  | 1 | Yes | Yes | No |
| 9 | AD | - | CDDP+VNR+RT |  |  |  |  |  | 1 | Yes | No | No |
| 10 | AD | EGFR | afatinib |  |  |  |  |  | 1 | No | Yes | No |
| 11 | LCNEC | - | CDDP+CPT-11 | Nivo | CBDCA+VP16 | Nivo |  |  | 4 | Yes | No | Yes |
| 12 | Sarcomatoid | - | CBDCA+nabPTX | Nivo |  |  |  |  | 2 | Yes | No | Yes |
| 13 | AD | - | Pembro |  |  |  |  |  | 1 | No | No | Yes |
| 14 | AD | - | CBDCA+PEM+Pembro |  |  |  |  |  | 1 | Yes | No | Yes |
| 15 | AD | - | Pembro |  |  |  |  |  | 1 | No | No | Yes |
| 16 | AD | ROS1 | CDDP+PEM+BEV |  |  |  |  |  | 1 | Yes | No | No |
| 17 | AD | EGFR | afatinib |  |  |  |  |  | 1 | No | Yes | No |
| 18 | AD | EGFR | CDDP+VNR | osimertinib |  |  |  |  | 2 | Yes | Yes | No |
| 19 | SQ | - | CBDCA+nabPTX |  |  |  |  |  | 1 | Yes | No | No |
| 20 | AD | ROS1 | entrectinib |  |  |  |  |  | 1 | No | Yes | No |
| 21 | AD | - | CBDCA+PEM+Pembro+GK |  |  |  |  |  | 1 | Yes | No | Yes |
| 22 | AD | EGFR | osimertinib |  |  |  |  |  | 1 | No | Yes | No |
| 23 | SQ | - | CBDCA+PTX |  |  |  |  |  | 1 | Yes | No | No |
| 24 | AD | EGFR | gefitinib | Pembro | CBDCA+PEM+BEV |  |  |  | 3 | Yes | Yes | Yes |
| 25 | SQ | - | CBDCA+PTX |  |  |  |  |  | 1 | Yes | No | No |
| 26 | AD | EGFR | osimertinib |  |  |  |  |  | 1 | No | Yes | No |
| 27 | SQ | - | CBDCA+nabPTX+GK | S-1 |  |  |  |  | 2 | Yes | No | No |
| 28 | AD | - | CBDCA+PTX | CBDCA+PEM+IPI+Nivo |  |  |  |  | 2 | Yes | No | Yes |
| 29 | SQ | - | CDDP+VNR | CBDCA+PTX | Durva |  |  |  | 2 | Yes | No | Yes |
| 30 | AD | RET | CBDCA+PEM+Pembro | Serpecatinib |  |  |  |  | 2 | Yes | Yes | Yes |
| 31 | Pleo | - | Pembro |  |  |  |  |  | 1 | No | No | Yes |
| 32 | SQ | - | CBDCA+PTX+Pembro | CDDP+VNR+RT | Pembro |  |  |  | 3 | Yes | No | Yes |

AD, Adenocarcinoma; BEV, Bevacizumab; CBDCA, Carboplatin; CDDP, Cis-Diammine-Dichloro-Platinum; CPT-11, Irinotecan Hydrochloride; DOC, Docetaxel; Durva, Durvalumab; GEM, Gemcitabine; GK, Gamma Knife; ICI, Immune Checkpoint Inhibitor; IPI, Ipilimumab; LCNEC, Large Cell Neuroendocrine Carcinoma; nabPTX, nanoparticle albumin-bound Paclitaxel; Nivo, Nivolumab; Pembro, Pembrolizumab; Pleo, Pleomorphic Carcinoma; PTX, Paclitaxel; RT, Radiotherapy; Sarcomatoid, Sarcomatoid Carcinoma; SQ, Squamous Cell Carcinoma; VNR, Vinorelbine; VP-16, Etoposide; WBRT, Whole Brain Radiotherapy

**Table S2. Perioperative Outcomes and Complications**

| Patient No. | Surgical Approach | Op Time (min) | Blood Loss (ml) | Hospital Stay (days) | Clavien–Dindo Grade | Complication Details |
| --- | --- | --- | --- | --- | --- | --- |
| 1 | open | 165 | 40 | 10 | 0 | none |
| 2 | open | 284 | 2500 | 21 | 3 | Prolonged Air Leak |
| 3 | open | 333 | 560 | 6 | 0 | none |
| 4 | open | 253 | 90 | 7 | 0 | none |
| 5 | open | 216 | 230 | 8 | 0 | none |
| 6 | open | 227 | 10 | 11 | 0 | none |
| 7 | open | 195 | 40 | 8 | 0 | none |
| 8 | open | 222 | 130 | 9 | 0 | none |
| 9 | open | 260 | 20 | 14 | 2 | Pain |
| 10 | VATS | 119 | 0 | 2 | 0 | none |
| 11 | open | 170 | 30 | 4 | 0 | none |
| 12 | open | 205 | 120 | 6 | 0 | none |
| 13 | open | 342 | 280 | 6 | 0 | none |
| 14 | VATS | 227 | 0 | 2 | 0 | none |
| 15 | VATS | 197 | 100 | 2 | 0 | none |
| 16 | VATS | 58 | 1 | 2 | 0 | none |
| 17 | VATS | 179 | 2 | 3 | 1 | Recurrent Nerve Palsy |
| 18 | VATS | 227 | 5 | 3 | 0 | none |
| 19 | VATS | 226 | 30 | 2 | 0 | none |
| 20 | VATS | 267 | 40 | 2 | 0 | none |
| 21 | VATS | 176 | 3 | 2 | 0 | none |
| 22 | VATS | 264 | 3 | 2 | 0 | none |
| 23 | open | 285 | 70 | 5 | 0 | none |
| 24 | open | 177 | 15 | 5 | 0 | none |
| 25 | VATS | 314 | 125 | 8 | 2 | Af |
| 26 | VATS | 253 | 30 | 3 | 0 | none |
| 27 | open | 253 | 150 | 6 | 0 | none |
| 28 | open | 140 | 10 | 4 | 0 | none |
| 29 | open | 265 | 30 | 6 | 0 | none |
| 30 | open | 155 | 5 | 5 | 0 | none |
| 31 | open | 345 | 410 | 9 | 0 | none |
| 32 | VATS | 180 | 5 | 5 | 0 | none |

VATS, Video-Assisted Thoracoscopic Surgery

**Table S3. Adjuvant Therapies and Post-recurrence Therapies**

| Patient No. | Adjuvant Therapy | Therapy after Rec. 1 | Therapy after Rec. 2 | Therapy after Rec. 3 | Therapy after Rec. 4 | Therapy after Rec. 5 | Therapy after Rec. 6 |
| --- | --- | --- | --- | --- | --- | --- | --- |
| 1 | Gefitinib |  |  |  |  |  |  |
| 2 | none | CBDCA+GEM | DOC | Nivolumab |  |  |  |
| 3 | none |  |  |  |  |  |  |
| 4 | none |  |  |  |  |  |  |
| 5 | none | Alectinib | CBDCA+S1 | CBDCA+nabPTX | Brigatinib | Lorlatinib | Brigatinib |
| 6 | Erlotinib | Osimertinib |  |  |  |  |  |
| 7 | none | Osimertinib | Osimertinib |  |  |  |  |
| 8 | none | DOC | Gefitinib | nabPTX | Afatinib |  |  |
| 9 | none |  |  |  |  |  |  |
| 10 | Afatinib |  |  |  |  |  |  |
| 11 | Nivolumab | AMR | Atezolizumab | Pembrolizumab |  |  |  |
| 12 | none | Surgery+SRT |  |  |  |  |  |
| 13 | none |  |  |  |  |  |  |
| 14 | none | CBDCA+PTX | Durvalumab | DOC+Ramucirumab |  |  |  |
| 15 | CBDCA+PEM+Bev+Atezo |  |  |  |  |  |  |
| 16 | PEM |  |  |  |  |  |  |
| 17 | Afatinib | Osimertinib | CBDCA+PEM | nabPTX | Osimertinib | CPT-11 |  |
| 18 | Osimertinib | RT |  |  |  |  |  |
| 19 | none | Surgery |  |  |  |  |  |
| 20 | Entrectinib | CBDCA+PEM+Pembro |  |  |  |  |  |
| 21 | none | CBDCA+PEM+Pembro |  |  |  |  |  |
| 22 | Osimertinib |  |  |  |  |  |  |
| 23 | none |  |  |  |  |  |  |
| 24 | none | Osimertinib |  |  |  |  |  |
| 25 | CDDP+VNR |  |  |  |  |  |  |
| 26 | none |  |  |  |  |  |  |
| 27 | none |  |  |  |  |  |  |
| 28 | Nivolumab |  |  |  |  |  |  |
| 29 | none | Pembrolizumab |  |  |  |  |  |
| 30 | Selpercatinib |  |  |  |  |  |  |
| 31 | none | Gamma knife | CBDCA+nabPTX |  |  |  |  |
| 32 | none |  |  |  |  |  |  |

AMR, Amrubicin; Atezo, Atezolizumab; Bev, Bevacizumab; CBDCA, Carboplatin; CDDP, Cisplatin; CPT-11, Irinotecan Hydrochloride; DOC, Docetaxel; DOC, Docetaxel; GEM, Gemcitabine; nabPTX, nanoparticle albumin-bound Paclitaxel; PEM, Pemetrexed; Pembro, Pembrolizumab; PTX, Paclitaxel; Rec, Recurrence; SRT, Stereotactic Radiotherapy; VNR, Vinorelbine

**Fig. S1. Distribution of Recurrence Patterns After Salvage Surgery**


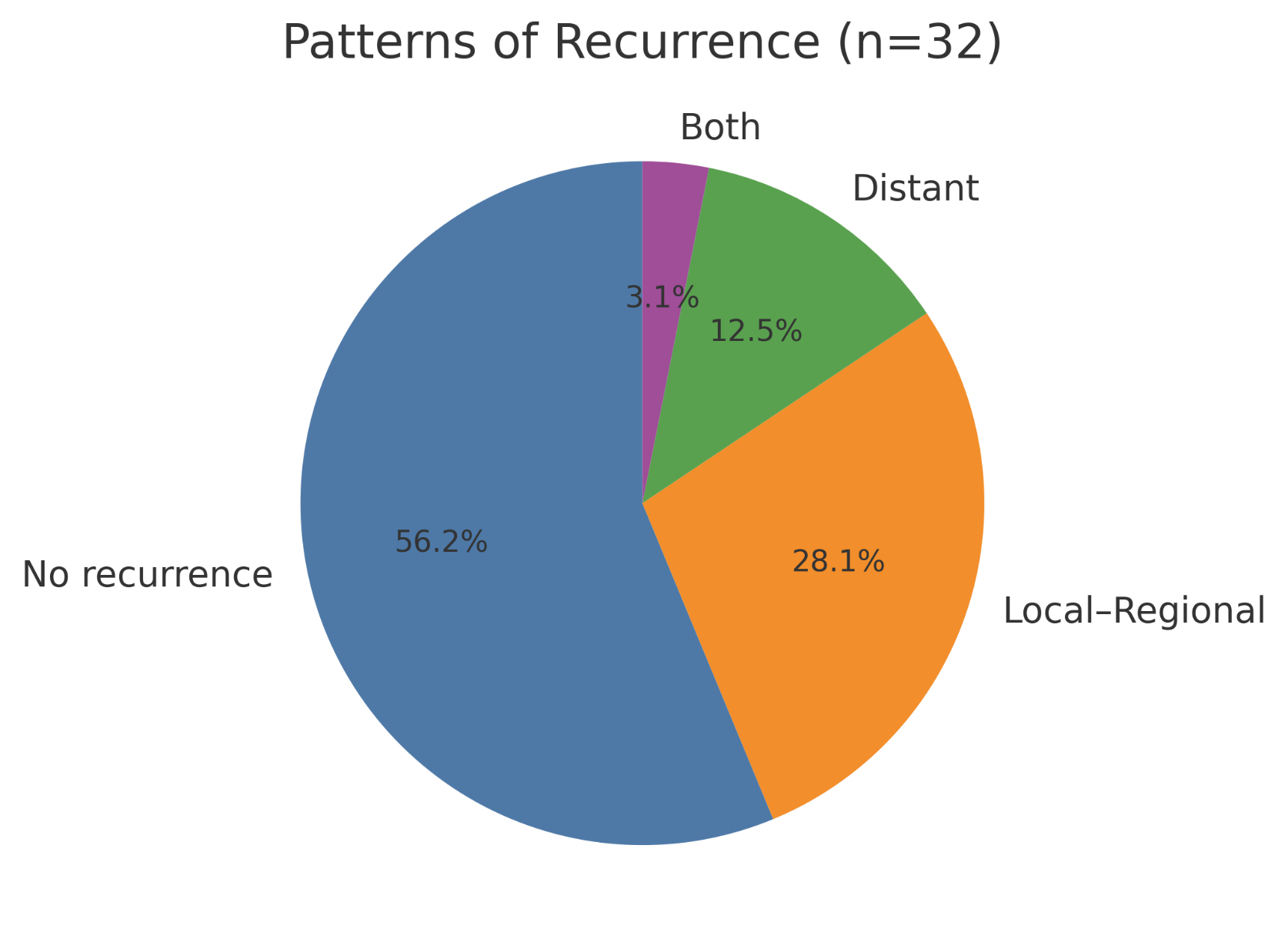


Pie chart of recurrence patterns observed after salvage surgery. The distribution is categorized into four groups: No recurrence, Local / Regional, Distant, and Both (local + distant). Percentages are calculated on a per-patient basis and displayed within the chart.
